# Supplementary material for: Are carbon emissions trading and green financial instruments synergistic? -Comprehensive quantitative research based on content analysis
Source: PLoS One. 2024 Mar 7;19(3):e0298601. doi: 10.1371/journal.pone.0298601 (PMC10919601; doi:10.1371/journal.pone.0298601)
Supplement: S1 Data — (ZIP) [file pone.0298601.s001.zip › Data/地级市Z_F工作报告.zip!/┴╔─■/░░╔╜╩╨/└┤╫╘╣1⁄2╓┌║┼í╛┬φ┐╦╩2╛▌═°í┐_2004.docx]

各位代表：

现在，我代表市人民政府向大会报告工作，请予审议，并请政协各位委员和列席人员提出意见和建议。

一、二○○三年政府工作回顾

2003年是本届政府任期的第一年。在市委的领导下，我们高举邓小平理论伟大旗帜，坚持以“三个代表”重要思想为指导，全面贯彻党的十六大精神，以开放促改革、促调整、促发展，全力推进经济建设和社会事业发展，较好地完成了市十三届人大一次会议确定的工作任务。国民经济步入加速发展的快车道，各项社会事业全面进步，城乡人民生活水平有了新的提高。特别是面对突如其来的非典疫情，坚持“两手抓”，取得了抗击非典和经济社会发展的双胜利。预计全年实现国内生产总值801亿元，比上年增长13．1％，达到10年来最好水平。完成财政一般预算收入28亿元，按可比口径增长13．3％。全社会固定资产投资达到175亿元，增长17．2％。据统计快报显示，鞍钢完成销售收入310亿元、增加值111亿元，实现了历史性跨越，为全市经济发展做出了重大贡献。

全面扩大对外开放，经济发展后劲明显增强。紧紧抓住项目这个核心不动摇，通过承办第二届台湾知名企业家辽东半岛观光周和举办香港推介会等一系列招商活动，台塑南亚、北美集团等一批知名企业相继来鞍投资。滚动推出139个亿元以上项目，其中103个项目进入实施阶段。投资15亿美元的海城纺织工业园前期工作基本就绪，其中投资3亿美元的差别化纤维项目已获国家批准。欧文斯科宁PVC外墙挂板、台湾中橡2万吨软质炭黑生产线、三和轧钢年产30万吨重型汽车用钢等17个重大项目建成投产。金山公司年产20万吨镀锌板和15万吨彩涂板、城市集中供热等10个投资5亿元以上项目开工建设。鞍钢新2＃高炉、后英集团大豆深加工等4个项目被国家列入东北老工业基地调整改造第一批国债专项计划。全年实际利用外商直接投资2．2亿美元，比上年增长34．1％；争取到位内资45亿元，增长20％。实现出口创汇4亿美元，增长13．3％。

全力推进农业产业化，农业基础地位进一步巩固。积极发展专用玉米、高油大豆、优质水稻等10个特色产品，建设了4个省级、6个市级和78个县乡级农业产业园区。订单农业发展到90万亩，比上年增长28．6％。无公害农业达到35万亩，占总播种面积的10．3％。动物无规定疫病示范区建设通过国家验收。开工建设了30万吨大米及副产品深加工、完达山10万吨液态奶等一批龙头项目，全市龙头企业发展到378家。“公司＋基地”带动农户面达到50％。粮豆总产量108．6万吨，预计实现农业增加值55．5亿元，比上年增长6．4％，畜牧业产值占农业比重达到52％。完成植树造林作业面积41．2万亩，增长36％。新增灌溉面积7．8万亩，改善涝区11．1万亩。农村税费改革全面推进，减少行政村309个，撤并学校175所，农民人均负担由152元下降到65元，减负率达57％。

进一步深化国有企业改革，经济运行质量明显提高。推广“两啤一化”模式，对客车厂等5户企业实施了脱胎换骨式改造。完成33户省考核国有企业改制任务，对60户企业依法实施了破产。选择11个项目，进行了财政建设性国有资产有效流动和退出的探索。大力支持鞍钢改革和发展，财政出资8000万元帮助鞍钢解决500万吨精品板材项目用地问题，冀东水泥熟料等地企合作项目开工建设。加快发展民营经济，宝得钢铁公司100万吨钢、鞍轮集团120万套全钢丝子午线轮胎等一批大项目开工建设，民营经济对国内生产总值增长的贡献率达到72．6％。预计全年规模以上工业实现增加值190亿元，比上年增长17．6％；实现销售收入585亿元，增长16．6％；实现利润30亿元，增长53％。

努力提高科技创新能力，高新技术产业迅速发展。承办了辽宁省省院校合作工程项目洽谈会，与清华大学、大连理工大学等院校签订合作协议80项。举办了海外学子创业周活动，签订技术引进和项目合作协议50项。纳米粉体制备技术等3个项目列入国家863计划，实现历史性突破。建成了聚龙、海诺、华冶等企业研发中心。全市省级高新技术企业发展到142家，实现高新技术产品产值103亿元，比上年增长41．9％。科技对经济增长的贡献率达到48％。高新区发展又迈出新步伐，引进了华融混合动力客车、清华同方环保工业园、北科大智能化控制同步开关高压断路器、吉兆公司数字电视广播发射设备等一批重点项目。全年实现国内生产总值39亿元，增长15％；实际利用外商直接投资3010万美元，增长20％。

积极克服非典影响，第三产业保持较快增长。对非典期间受到较大冲击的旅游、餐饮等服务行业，全面落实国家出台的扶持政策，适时启动商贸和旅游市场。开工建设了大福源超市、中海物流等一批大型现代化商业设施，引进了国美电器、百世特等大型连锁企业，新增各类超市80家。千山风景区首次被列为全国黄金周假日旅游预报景区，千山和玉佛苑被评为全国知名旅游品牌。玉佛苑二期工程、北方梅园等旅游项目正式启动。预计全年实现第三产业增加值307亿元，比上年增长13．5％；实现社会消费品零售总额171亿元，增长12．6％。全年接待国内旅游者570万人次、境外旅游者4．6万人次，实现旅游综合收入40亿元，增长5％。

认真做好财税金融工作，基本化解了“两债”兑付风险。建立了市县两级分税制财政体制，完善了与之配套的财政转移支付制度。按照建立公共财政体制框架的要求，试行了部门预算和国库集中收付制度，加强了政府采购和政府债务管理工作，保证了财政资金的合理使用。坚持依法治税，严格税收征管，做到了应收尽收。各金融机构不断调整信贷结构，积极支持经济建设，不良贷款比例压缩2个百分点，为破产企业核销呆账19．6亿元。积极争取国家和省的大力支持，有序开展了鞍山证券、海城信托撤销后的兑付工作和五环证券撤销后的登记、确认工作，全市“两债”余额由年初的16．2亿元降至2．7亿元，下降83．3％。

不断加强城乡建设与管理，投资环境进一步改善。城乡面貌发生新的变化，城市的现代化水平又上新台阶。建成了城市规划展示厅、国际会议中心，完成了客运站主体工程，启动了站前商业街改造和南沙河综合整治工程。全面完成了市区内主干道的大修改造，鞍羊线和鞍下线鞍山段及海城外环全线建成，新修村村通油路314公里。建设城镇住宅150万平方米，改造居民小区56个，在50％的老社区实施了开放式物业管理。城镇栽植大树9万株，市区栽植乔灌木167万株，新增城市绿地139．8公顷，进入省园林城市行列。完成矿山植被恢复和防风固沙林1164公顷，新增自然保护区面积5600公顷。玉佛山风景区内占压建筑清理完成64％。羊耳峪垃圾处理场沼气利用二期工程投入试运行。实施5项水污染治理工程和9项大气环境治理工程，杨柳河结束“红河”历史，取缔4吨以下燃煤锅炉140台，城市空气质量全年有62％天数达到国家二级标准。继续保持了国家卫生城和道路畅通工程A类一等模范城市称号。顺利实施了城市供暖收费体制改革。对经济环境进行了集中整治，行政审批事项由462项精简到397项，构建了市场准入和建筑工程项目审批两条绿色服务通道。

深度推进社会保障和就业工作，人民生活水平稳步提高。圆满完成了省部署的社会保障试点工作任务。基本养老、医疗保险覆盖面分别达到100％和80．2％，失业保险扩面2．5万人。在城区35个街道、9个乡镇及327个社区全部设立了劳动保障工作机构，有50％社区的硬件设施达到省规定标准，失业人员和地方企业离退休人员已全部纳入社区管理。建成了城市低保信息管理网络，低保对象达到9．2万人，月人均救助额提高到75元。以创业促进就业，全年兴办再创业企业305户，“孵化”再创业企业86户，多渠道实现就业总量11．2万人次。积极开展扶贫帮困活动，全年筹集款物7000万元，为“三老”和贫困户建房420户，包户扶持1．8万户，脱贫率50％以上。预计全年城市居民人均可支配收入达到7430元，比上年增长8．5％；农民人均纯收入达到4000元，增长5．8％。

切实加强精神文明建设，社会各项事业全面进步。贯彻《公民道德建设实施纲要》，开展了以“打造诚信鞍山、培育诚信公民”为主题的诚信教育和实践活动，进一步塑造了“鞍山在我心中、我为鞍山奉献、鞍山为我自豪”的鞍山人精神。强化行风建设，全市重点窗口行业社会综合满意率达到85％。深入开展城乡精神文明共建活动，巩固了创建全国文明城市工作成果。大力推进素质教育，强化了高中教育和职业教育，全市高考综合录取率达到92％，比上年提高3个百分点。积极整合教育资源，进一步改善了办学条件，着力解决了教育热点问题。完成了博物馆和西苑古玩市场等文化设施建设的前期工作。通过市场化运作，建成了数码电影城主体工程。组建了鞍山市艺术学校，推进了专业表演艺术团体的改革。成功组织非典防治工作，安排3110万元财政专项资金，完善了公共卫生体系，建立了公共安全和公共卫生应急处理机制，实现了非典零病例和无疫区的目标。巩固军政军民关系，第三次蝉联全国“双拥”模范城市称号。人口与计划生育、广播电视、新闻出版、体育、民族、宗教、外事、侨务、接待、妇女儿童、国防教育、民兵、预备役、人防、残联、统计、档案、史志、老龄、气象、地震等工作也都取得新的成绩。

进一步加强民主法制建设，树立了执政为民的政府形象。坚持重大事项及时向人大及其常委会报告和与人民政协进行民主协商制度，自觉接受人大依法监督、政协民主监督和人民群众社会监督。深入开展“四五”普法教育，人民调解、安置帮教、法律援助在化解社会矛盾中发挥了重要作用。积极推进依法行政，提请市人大审议地方性法规2件，颁布政府规章6件。完善政府公告制度和法律顾问制度，提高了政府决策的民主化和科学化水平。树立正确的政绩观，坚持群众利益无小事，密切了与人民群众的血肉联系。人大代表建议和政协委员提案办结率100％，直播《今晚会见》节目45期，受理群众来信来访6．8万件，受理市民投诉2．9万件，批办市长信箱电子信件482件，有效解决了影响改革、发展和稳定的热点难点问题。深入开展行政监察工作，严肃查处了一批违法违纪案件。强化社会治安综合治理，严厉打击了各种刑事犯罪，维护了公共秩序和社会稳定。

各位代表，经过几年的努力，鞍山国民经济和社会发展已经步入快速增长时期，全市广大干部群众求发展、盼振兴的热情高涨，民气大增，人气正旺。这是市委正确领导及人大依法监督、政协民主监督的结果，是全市各族人民辛勤劳动、共同努力的结果，也是鞍钢等中省直单位、驻鞍部队和海内外朋友大力支持的结果。在此，我代表市政府全体成员，向全市人民及所有关心和支持鞍山发展的各界朋友，致以衷心的感谢和崇高的敬意！

在肯定成绩的同时，我们也清醒地看到全市经济和社会发展中的深层次矛盾还没有根本解决，在发展过程中也出现了一些新情况和新问题。一是对外开放的深度还不够，领域还不宽，利用外资占投资总量的比重仍然较小。二是经济增长方式还比较粗放，资源浪费大、能耗高、环境污染等影响可持续发展的问题远未解决。三是结构调整步伐还不大，市场化、产业化程度还不高，民营经济发展还不够快。四是就业压力较大，农民收入增长缓慢，部分群众生活还比较困难，社会不稳定因素仍然较多。五是经济发展的软环境仍不够宽松，法制环境、服务环境、人文环境等方面还存在诸多问题。一些部门的服务意识和服务能力还不强，有的部门工作人员仍然存在“不作为”和“乱作为”现象。这些问题，我们已经高度重视，并将在今后工作中用改革和发展的办法，努力加以解决。

二、全面推进鞍山老工业基地调整改造和振兴

党中央、国务院关于支持东北地区等老工业基地调整改造和振兴的重大战略决策，为鞍山加速发展提供了千载难逢的历史机遇。根据市委九届七次全会做出的战略部署，市政府编制了《鞍山老工业基地调整改造和振兴规划（草案）》，并提交这次大会审议。我们确定鞍山老工业基地调整改造和振兴的指导思想是：以“三个代表”重要思想为指导，全面贯彻党的十六大和十六届三中全会精神，牢固树立全面、协调、可持续的发展观，实施工业强市方略，以结构调整为主线，以改革开放和科技进步为动力，以壮大支柱产业、做强骨干企业、扩大社会就业、完善社保体系、改善发展环境为重点，用新思路、新体制、新机制、新方式，走出老工业基地调整改造和振兴的新路子，实现国民经济持续快速协调健康发展和社会全面进步。到2010年，基本建成全国重要精品钢材基地，基本完成老工业基地振兴任务，基本实现工业化。在优化结构和提高效益的基础上，国内生产总值年均增长13％以上，实现经济总量提前翻番，综合经济实力进入省内第一集团；形成比较完善的现代市场体系、社会保障体系、政策法规体系和公共行政服务体系，建立起更具活力、更加开放的经济体系；实现城市与农村、经济与社会、人与自然协调发展，为全面建设小康社会奠定坚实基础。

按照上述指导思想和奋斗目标，振兴鞍山老工业基地的战略任务和主要措施有以下六个方面：

（一）以建设“一个基地、三大产业”为重点，构筑现代产业格局，实现产业结构优化升级。实施工业强市方略，建设全国重要的精品钢材基地，壮大先进装备制造、轻纺和矿产品加工三大支柱产业，以工业化带动经济全面发展，形成以现代农业为基础、高新技术产业为先导、工业为主体、服务业全面发展的新型产业结构。以鞍钢为依托，壮大以汽车板、家电板、造船板、新型建筑用钢为重点的钢铁产业群，构筑鞍钢经济圈。到2010年，全市钢和钢材的综合生产能力分别达到2000万吨和2200万吨。以特种机械和专用设备为主攻方向，运用高新技术和先进适用技术改造提升装备制造业，重点培育冶金设备、矿山设备、工程机械、改装汽车、环保设备和智能化设备等系列产品。以海城纺织工业园为龙头，开发中高档纺织产品以及特色食品，成为国内重要的轻纺产品集散地。以提高资源综合利用率为目标，做深做精矿产品加工业，推进二次资源开发，形成具有竞争优势的铁精矿、高纯镁砂、高档滑石粉、精品玉器等一批拳头产品。以增强技术创新能力为手段，加快电子信息、精细化工、环保、新材料等高新技术产业化进程，建设国内一流的高新技术产业开发区。用工业化理念谋划农业发展，实现粮食、蔬菜、水果、畜牧等农产品的产业化生产和规模化经营，形成具有鞍山特色的现代农业发展模式。以旅游业为龙头，大力发展现代服务业和其他新兴第三产业，把鞍山建设成为中国北方优秀旅游名市和辽南中部地区商贸物流中心。

（二）以引进大项目为主攻方向，强力推进招商引资，构建开放型地区经济格局。坚持以开放促改革、促调整、促发展，全面建设开放型地区经济的战略构想，以项目为核心，主动将鞍山融入世界新一轮产业调整之中，在更加开放的环境中推进老工业基地的全面振兴。紧跟世界产业转移步伐，积极承接韩国、新加坡、日本、美国以及台湾、“长三角”、“珠三角”等国家和地区加工制造业的转移。积极融入沈阳经济区，主动接轨沈大经济带，加强区域间分工协作，实现优势互补和错位发展。创新招商组织方式，以世界500强、跨国公司、国内知名大企业、上市公司和著名民营企业为重点，积极开展小团组招商、中介招商和网络招商，每年滚动实施投资亿元以上项目不少于200个。扩大开放领域，实现金融、旅游等现代服务业及公用事业、教育、文化、卫生等领域利用外资新突破。努力扩大机电产品、轻纺产品、农产品和成套设备出口，实现出口增长高于经济增长。实施“走出去”战略，扩大对外交流与合作，开展境外加工贸易和工程承包，全面提升鞍山的国际化水平。

（三）以增强地区经济活力为目标，培育和规范市场主体，建立完善的社会主义市场经济体制。落实十六届三中全会精神，按照社会主义基本经济制度要求，积极调整国有经济布局，形成国有经济以鞍钢为主体、地方经济以民营为主体的发展格局。2004年全部完成地方国有工业企业改制退出任务。力争用两到三年时间，支持鞍钢完成辅业分流和集体企业改制任务。基本完成公用事业、文化、体育等财政建设性国有资产的有效流动，实现地方国有资本从一般性竞争领域全部退出。大力推进公司制改造，全面建立现代企业制度。彻底清理限制民营经济发展的政策规定，营造鼓励投资创业的社会氛围，实现民营经济的大发展。调整部门职能，理顺各种关系，建立行为规范、运转协调、公正透明、廉洁高效的行政管理体制。健全国有金融资产，非经营性资产和矿产资源的监管体系，建立权责明确、管理规范、上下协调的国有资产管理体制。打破地域界限，培育生产要素市场，发展各类专业市场，树立规则意识，规范中介行为。形成公平竞争、统一开放的市场体系。进一步健全养老、失业、医疗等社会保险制度和城镇居民最低生活保障制度，着力改善就业环境，建立起完备的社会保障体系和社会就业机制。

（四）以规划建设“两园一区一带”为龙头，调整区域经济布局，实现城乡协调发展。围绕提高产业聚集效应和提升综合竞争力，重组城乡发展空间和产业布局。在鞍钢以西、千山西路以北、沈大高速公路以东的53平方公里范围内，建设鞍山工业园，重点发展冶金、机械、化工、建材等传统产业，承接招商引资项目和城区企业的搬迁改造。以高新区现有东、西两园为基础，扩充项目用地，做大高新技术产业园。以鞍钢现有厂区为主体，向西扩展3．2平方公里，拓展鞍钢生产加工区。以城市新区建设为龙头，规划建设鞍海新经济带。同时，以资源、区位、市场和产业优势为依托，加快海城经济开发区、腾鳌经济开发区及各类工业小区的规模化和特色化建设，全面整合城乡资源，打破二元结构，加速农村工业化和城镇化进程，形成区域经济发展新格局。

（五）以创建全国文明城市为载体，促进社会事业全面进步，实现人的全面发展。坚持以人为本，牢牢把握先进文化的前进方向，传承具有厚重历史、培育具有时代特征、发掘具有地域特色的城市文化，提高人们的科学文化素质、民主法律意识和思想道德水平。实施科教兴市战略和人才强市战略，加快科技创新体系建设，广泛开展校企合作，强化高新技术的引进和推广，开发具有自主知识产权的高新技术产品，到2010年科技进步对经济增长的贡献率达到60％以上。积极创新教育体制，在强化基础教育的同时，大力发展职业技术教育，不断优化高中等院校的专业设置，培养适应产业结构优化升级需要的复合型人才和实用型人才，全面提高劳动者的创业能力和就业技能。深化文化体制改革，形成先进的文化体制、发达的文化产业和浓厚的文化氛围。加强公共卫生工作，健全疾病预防控制体系和医疗救治体系。建立和完善各种应急机制，形成覆盖全市的社会管理体系和社会服务体系。

（六）以优化创业和人居环境为切入点，加强民主法治建设，进入国内投资环境最佳城市行列。按照政治生活民主化、经济生活规则化、社会生活法治化的发展要求，努力营造与社会主义市场经济体制相适应的民主政治和社会环境。加强民主政治建设，推进政府决策的民主化和科学化，建设社会主义政治文明。认真实施《中华人民共和国行政许可法》，完善公共行政服务体系，构筑适应市场经济发展、符合WTO规则的绿色服务通道。建立个人信用体系，搭建企业联合信用平台，树立诚信政府形象，形成以道德为支撑、产权为基础、法律为保障的社会信用制度，打造“诚信鞍山”。积极推进依法治市，建立权责明确、行为规范、保障有力的法律执行体系和治安防范体系。不断加强城乡基础设施建设，全面改善群众的生产生活条件。依法加强对水、耕地、矿产等资源的管理，大力推广新型建筑材料，实现自然资源的合理开发和永续利用。以河流污染整治、空气污染治理和造林绿化为重点，全面改善生态环境，进入国家环保模范城市和国家园林城市行列。

各位代表，振兴鞍山老工业基地是历史和人民赋予我们的重大责任。我们要不辱使命，不负重托，自强不息，团结奋进，以饱满的工作热情和顽强的拼搏精神，打赢这场关乎鞍山兴衰、关乎人民福祉的伟大战役，再造祖国钢都新的卓越与辉煌，谱写鞍山发展新的历史篇章。

三、二○○四年经济和社会发展主要任务

2004年是全面推进鞍山老工业基地调整改造和振兴的关键一年。根据中央、省和市委的战略部署，特别是按照市委九届七次全委会议的要求，今年全市经济和社会发展的总体安排是，认真组织鞍山老工业基地调整改造和振兴规划的实施，坚持以开放促改革、促调整、促发展，以项目为核心，以结构调整为主线，内抓放开，外抓对接，创新增量，协调发展，用新思路、新举措、大项目，形成加速发展、加快振兴的新局面，确保实现高起点快起步开局、高水平跨越式发展。主要预期目标是，实现国内生产总值增长13％，财政一般预算收入增长13．6％，全社会固定资产投资增长16％，城市居民人均可支配收入增长8％，农民人均纯收入增长5％。

（一）全力推进产业结构调整，提升工业经济整体素质

壮大支柱产业。围绕建设“一个基地、三大产业”，实施“双百工程”，培育和扶持100户重点工业企业，规划和实施100个投资5000万元以上的重点工业项目。钢铁工业，重点支持鞍钢加快500万吨精品板材和三冷轧110万吨冷轧板等项目建设；促进三冶60万吨精品焊管、鞍钢实业40万吨钢渣综合利用等项目建设。地方重点推进西洋集团300万吨精品钢材、后英集团100万吨海绵铁、金山公司20万吨镀锌板及15万吨彩涂板、鞍特公司10万吨重型钢结构等项目开工建设；加快宝得钢铁公司100万吨钢二期工程、衡业公司60万吨汽车用钢等项目前期工作。装备制造业，推进海诺集团3000辆混凝土搅拌运输车及100台臂式泵车、衡业集团5000辆改装专用汽车及载重汽车、华融公司1000辆混合动力客车等项目开工建设；加快鞍钢机械制造公司冶金成套设备、电子电力公司5000套光纤传感器等项目前期工作。轻纺工业，重点抓好海城纺织工业园中的20万吨差别化纤维等6个项目开工建设；促进完达山年产10万吨液态奶一期工程、后英集团30万吨大豆深加工、椰风集团3万吨罐头饮料等项目竣工投产；推进台塑南亚6万吨PVC管材、味邦集团年产5万吨有机酱油等项目开工建设；加快天海服装高档纺织及服装生产线、阿迈特公司2000吨玻璃钢管等项目前期工作。矿产品加工业，促进鑫荣集团年产2万吨耐火材料、福海公司3万吨高性能滑石系列母粒等项目竣工投产；推进海城中兴集团与中科院合作年产5万吨镁合金制品、岫岩偏岭镇与江苏苏嘉集团合资生产镁碳砖等项目开工建设；加快绿鑫佳美公司年产60万吨钾镁肥料、泛亚公司电熔镁等项目前期工作。

加快发展高新技术产业。健全和完善数字化制造联合技术中心，抓好信息化技术在冶金、化工、纺织、机械、新材料等五大行业的应用。壮大数字电视发射装置、电气及自动化装备、特种工程机械等高科技产业，做大金融机具、环保、精细化工产业园。加快鞍山科大纳米粉体制备技术、模拟移动床等科研成果的产业化进程。省级高新技术企业达到152户，实现高新技术产品产值120亿元。

加快园区建设。积极启动鞍山工业园建设，引导中心城区企业实施“退二进三”搬迁改造。创新高新区管理体制，组建资产经营及投融资有限责任公司，实现高新区基础设施建设的市场化运作。开工建设科技广场，建成高新大厦主体工程。推进海诺二期等项目建设，引进亿元以上项目10个。建设海外学子产业化基地，新增进区创业项目50个。高新区全年实现国内生产总值增长25％，实际利用外商直接投资增长20％。

（二）加快完善基本经济制度，增强地区经济活力

调整国有经济布局。全面完成地方国有企业改制任务，基本实现国有资本从一般竞争性领域全部退出。以股份制为主要形式，按照“两啤一化”模式，对一工、北铸、矿机、冷弯、锅炉等10户地方国有大中型企业实施脱胎换骨式改造。依法对针织总厂新华印刷厂等30户劣势企业实施破产。按照现代企业制度要求，完善味邦集团等20户企业法人治理结构。

加快国有资产有效流动。坚持市场化原则，积极推进文化、体育、卫生、公益事业、科研院所等领域国有资产流动和退出，推进银座、钻石城、第二污水处理厂等项目签约，确保自来水公司、煤气公司、国际大酒店等项目取得实质性进展。

大力发展民营经济。发挥中小企业担保中心作用，设立中小企业发展专项资金，引导民间资本组建信用担保机构，拓宽民营企业融资渠道。指导建立民营企业协会和冶金、玉石、镁砂等行业协会。积极支持民营企业上市。扶持中兴集团、银海集团等一批企业建立技术中心。支持民营企业实施名牌战略，争创2个国家驰名商标、10个省著名商标和10个省部名牌产品。制定针对民营企业的奖励办法，对纳税大户给予必要的物质和精神奖励，在全社会营造纳税光荣和尊重纳税人的浓厚氛围。到年底，全市销售收入亿元以上民营企业达到55家，其中10亿元以上4家，20亿元以上2家。民营经济主要指标增长15％以上。

（三）深度推进地企融合，构筑鞍钢经济圈

大力支持鞍钢改革和发展。把鞍钢视为世界500强企业看待，为鞍钢生产建设提供全方位服务，重点在规划、用地等方面提供优惠政策。抓住国家支持东北老工业基地调整改造的历史机遇，全力帮助鞍钢争取国家扶持政策，做好企业办社会剥离工作，克服困难积极主动帮助鞍钢解决辅业改制分流和厂办集体企业问题。

努力拓展地方与鞍钢合作领域。支持鞍钢并购市第一轧钢厂、鞍山钢管集团以及与海城镁矿合作等项目建设，积极吸引鞍钢项目在地方注册。借助鞍钢优势，引导和发展钢铁精深加工，重点延伸建筑型钢、汽车用钢、工程焊管、镀涂薄板、精密带钢、应力线材等产业链条，壮大钢铁产业集群。引导地方企业深度开发鞍钢市场，积极为鞍钢提供配套产品，扩大鞍钢在地方的采购份额。

（四）广泛开展外引内联活动，切实引进一批大项目

加大招商引资工作力度。高水平谋划和组织鞍山台湾周、香港推介会等招商活动，积极争办WTO亚太成员公平贸易论坛等国内外重大会议，积极邀请各类专业投资团组来鞍考察访问，促进招商引资和项目对接。加强与台湾工商建研会、香港总商会、韩国贸易振兴协会、新加坡国企局等机构的紧密合作，拓展招商渠道。以小团组方式开展专业化对口招商，全年到境外招商不少于20次。强化对以温州为重点的长三角、以东莞为重点的珠三角地区招商工作，举办温州千人联谊会等招商活动。按注册资本计算，实际利用外商直接投资1．6亿美元。引进内资54亿元，增长20％。

强化项目滚动推出和推进机制。充实项目库，规划和实施一批能够促进跨越式发展的大项目。重点推进超亿元项目160个，其中新开工37个，续建81个，前期工作25个，策划17个，总投资600亿元，当年完成投资90亿元，比上年增长28％。力争洽谈签约一个相当于海城纺织工业园规模的大项目。继续做好国债项目的申报和争取工作。

千方百计扩大外贸出口。用足用好国家支持外贸出口政策，推进资源深加工产品、机电产品和高新技术产品进入国际市场，扩大农产品出口，争办出口加工区，确保全年出口创汇4．5亿美元。支持优势企业向国外拓展，开展对外工程承包，扩大劳务输出，积极参与国际竞争。

（五）继续强化农业基础地位，努力增加农民收入

进一步抓好“三农”工作。以发展民营经济为主，走农村工业化、农业产业化、农村城镇化的路子，加快发展县域经济。重点实施234个投资千万元以上项目。改革小城镇户籍管理制度，引导农村富余人员从事非农产业或外出务工，实现劳动力转移2万人。成立农民工咨询服务中心，下力量解决拖欠农民工工资问题。以县城建设为重点，加快农村城镇化步伐，新建村镇住宅50万平方米，完成村村通油路500公里，新增自来水受益人口3万人。

提升农业产业化水平。推广“公司＋农户＋基地”经营模式，通过土地流转、贷款贴息和财政补贴等手段，加快现代农业园区建设。积极组织实施沈大和沈山高速公路两翼开发，重点推进舒妍芦荟、万鑫茧丝、嘉禾绿色食品等10个超亿元产业化龙头项目建设。农事龙头企业发展到400户，“公司＋基地”带动农户面达到55％。实施农产品市场准入制度，大力发展绿色无公害农产品，壮大宁远等6个农副产品批发市场。

深化农村各项改革。进一步完善农村税费改革，全面落实各项财税政策，积极化解村级债务，推进乡镇事业单位机构改革，确保农民负担不出现反弹。按照依法、有偿、自愿的原则，完善农村土地流转制度。坚决遏制乱占耕地行为，保护基本农田。建立和完善农业科技推广体系、市场服务体系和农村社会化服务体系，大力发展农村合作经济组织，提高农民进入市场的组织化程度。按照国家部署，积极推进粮食流通体制改革。加大财政转移支付力度，改善农村信贷服务，支持农业基础设施建设和农村社会事业发展。落实党的民族政策，深入实施东部山区开发工程，确保62个重点贫困村脱贫工作完成省定目标。

加强农业基础设施和生态环境建设。增加节水灌溉面积2万亩，治理水土流失面积28万亩。加大森林资源保护，改善生态环境，抓好退耕还林、三北防护林、台安西北部沙漠化防治及海岫生态脆弱带恢复等项目建设，植树3000万株，造林20万亩。实施农村改水改厕工程，改善农民的生产和生活条件。

（六）加快发展第三产业，建立良好的市场经济秩序

壮大旅游经济。开展争创全国最佳旅游城市活动，加大旅游产品开发和宣传促销力度。完成旅游业发展规划的编制工作。重点推进中安达分时度假村、玉佛苑二期工程、千山滑雪场、乐雪绿色生态观光园等项目建设。引导和鼓励民间资本参与旅游项目和旅游纪念品开发。加强区域合作，打造东北地区旅游经典线路。扩充千山国际旅游节内涵，发挥国际商贸促进机构作用，实现旅游、商贸、会展三位一体发展。全年实现旅游综合收入50亿元，比上年增长25％。

发展现代服务业。实施商业发展规划，培育专业批发、餐饮服务等优势产业群，积极发展电子商务、连锁经营、物流配送等新型业态。大力发展市场中介、信息咨询、投资服务等现代服务行业。抓好味邦现代食品加工园、中海物流、钢材市场等5个超亿元项目。完成一百、寰球、天河等企业改制和国有股退出工作。

整顿市场经济秩序。严厉打击不正当竞争、欺诈经营、制假售假等扰乱市场秩序的违法行为，大力整顿和规范市场经济秩序，努力营造公平公正、诚实守信的市场环境。重点打击食品、药品、建材、汽车配件和农业生产资料等领域制假售假行为，营造安全放心的消费环境。加强税收征管，严厉打击各种偷税、逃税和骗税行为，做到应收尽收。发挥政府在社会诚信体系建设中的主导作用，启动企业联合信用平台建设，开展建立个人信用档案试点工作，加快健全社会信用体系。

深化投融资体制改革。放宽民间投资领域，激发民间投资热情，推进投资主体社会化和多元化。积极吸引各类金融机构入驻鞍山，出台鼓励金融机构支持地方经济发展的奖励政策。加大“两债”债权清收力度，降低银行不良贷款比例，提高银行资产质量，建设金融安全区。

（七）深度推进经营城市，提高城市建设管理水平

进一步完善城乡基础设施。做好城市新区规划编制工作，规划批准后，适时启动鞍山新区（汤岗子组团）建设。推进城市集中供热工程，开工建设引汤入鞍扩建及第二污水处理厂二期工程，做好轻轨项目的前期准备工作。完成站前商业区拆迁任务，年内开工建设。加快完善市区环路，建成深营路和兴盛路南延工程，打通深营路隧道。开展市郊大环路建设的前期工作。扩建东山隧道，改造千山西路，建成长途客运站、达道湾生活服务设施和台商会馆。新建城市住宅90万平方米。开通鞍山至北京航班。实施利民、便民、爱民工程，推进道路建设向小街小巷延伸，建设低收入群众安居工程，做好棚户改造收尾工作，全面完成市属居民小区改造和市区排水明渠改暗渠任务。协调鞍钢等中省直单位加快所属居民小区的改造步伐。

加大环境综合整治力度。巩固国家卫生城和畅通工程创建成果，深入开展国家环保模范城市和国家园林城市创建活动。完成玉佛山风景区占压建筑的清理工作，全面改造二一九公园，拓宽东山道路。完成9条道路绿化带建设和主干道两侧拆墙透绿工作，粉饰临街建筑100万平方米。加大城乡结合部环境卫生整治力度，清理整顿户外广告和占道市场，加快占道市场退路进厅步伐。全面完成地名设标任务，实现地名管理规范化。推进鞍钢化工总厂一期干熄焦工程等8个污染治理项目，取缔城市建成区4吨以下燃煤锅炉100台，加大机动车尾气治理力度，严格控制扬尘污染，城区空气质量全年65％天数达到国家二级标准。对重点污染企业和污染源进行集中整治，力争COD、烟尘防治提前一年完成省控目标。以海城五道河为重点，加强境内河流污染的综合整治。全面启动南沙河综合治理一期工程，停止宋三镇用污水灌溉水稻种植。完成矿山复垦100公顷，建设防风固沙林400公顷。

加强社区建设。开展市区领导、党政机关、企事业单位帮建社区活动，通过多种途径，使社区“一站四室”达到省规定标准。进一步改善社区办公条件，提高社区干部待遇，拓展社区服务领域，完善社区服务功能。在80％的城市社区实现规范的开放式物业管理。完成第三批“星光计划”。

（八）强化社会保障和劳动就业工作，进一步改善人民生活

继续完善社会保障体系。养老、失业和医疗保险各扩面1万人。完善社会保障信息网络，建立养老、失业保险金发放预警制度，确保各项社会保险费按时足额发放。加快医疗保险IC卡推广和普及使用，开展以大病统筹为主的新型农村合作医疗制度试点工作。加强对城镇特困群体的动态管理，实现低保对象的应保尽保。

努力扩大社会就业。实施就业优先发展战略，把促进就业作为全市“一号工程”，全面贯彻落实中央、省、市在税收、收费、财政、信贷等方面的再就业扶持政策，进一步做好《再就业优惠证》发放管理工作。实施增加就业岗位、增强就业技能的“双增工程”，拓宽就业渠道。建成再创业服务中心二期工程，加大对再创业企业的扶持力度，全年实现就业培训6万人，开发就业岗位9万个，城镇登记失业率控制在6％以下。

认真做好城乡扶贫帮困工作。推广“扶贫超市”，建立扶贫帮困长效机制，全年救助困难群众10万人次。开展扶贫帮困结对子活动，巩固包户扶持体系，包户扶持脱贫率达到30％以上。帮助贫困户建房200户。建立全市困难群众重大疾病医疗救助制度。

（九）加快发展社会各项事业，全面提高城乡文明程度

开展全国文明城市创建活动。坚持以人为本，深入贯彻落实《公民道德建设实施纲要》，加强社会公德、职业道德、家庭美德教育。深入开展创建文明社区、文明村镇、文明行业、文明单位及“共铸诚信”活动，倡导健康文明的生活方式。建设学习型城市，提升市民的思想道德素质和科学文化素质，提高社会文明程度。

实施人才强市战略。推进百千万人才工程、人才引进工程、村委人才工程等十大人才工程建设。以师德师风建设为重点，高水平、高质量普及九年义务教育。建设新一中校园，加强普通高中建设，推进普及高中阶段教育。创新教育管理体制，广泛吸引社会投资，大力发展职业技术教育，筹划鞍山冶金技术学院和鞍山外语学校。加快教育信息化建设，开播教育频道，启动“数字化校园”建设工程。

加快文化、卫生、体育等社会事业发展。推出一批优秀文艺作品，举办首届社区文化艺术节，加强档案馆、图书馆信息化建设，建成市博物馆。改革文化投资体制，推进演出团体市场化改革，规范文化市场秩序。巩固非典防治成果，健全公共卫生体系，完善突发性公共卫生事件应急防御体系。大力加强农村乡镇和城市社区卫生工作，建设10所农村中心卫生院。加大食品卫生监察力度，治理“餐桌污染”。开展大型公益性全民健身活动，提高竞技体育水平，推进体育产业市场化进程。加强安全生产和防火防盗工作，避免重大恶性事故发生，保障人民生命财产安全。按照国家统一部署，认真开展首次经济普查工作。大力开展国防教育，继续抓好民兵、预备役和人防工作，巩固“双拥”工作成果。全面做好人口与计划生育等各方面工作，促进经济和社会协调发展。

（十）加强政府自身建设，改善经济发展环境

推进民主与法制建设。继续坚持重大事项向人大及其常委会报告制度，积极支持人民政协履行政治协商和参政议政职能。自觉接受人大依法监督、政协民主监督，认真听取各民主党派、工商联、人民团体和无党派爱国人士意见。支持工会、共青团、妇联等群团组织有效开展工作。健全基层自治组织的民主制度，搞好第八届村委会换届选举工作。深入开展普法教育，提高全民特别是公职人员的法律意识。强化社会治安综合治理，全面推进警务改革，依法防范、扼制和打击各种犯罪活动，打造“平安钢都”。

认真组织开展环境建设年活动。以7月1日实施《中华人民共和国行政许可法》为契机，清理地方性法规、规章，规范政府行政行为。配套下放市级经济管理权限，扩大县（市）区在经济活动中的自主权和决策权。全面落实《中共鞍山市委关于进一步优化经济发展环境的意见》，完善公共行政服务体系，制定从根本上解决行政审批事项体外循环的措施。积极推进综合行政执法改革，强化对行政执法人员的监督检查，对影响投资环境、损害发展大局的行为严查重办。全力营造“办事不用求人、事事都有人管、说理必有地方”的投资环境，使鞍山成为省内软环境建设的典范地区。

切实改进工作作风。树立正确的政绩观，做到权为民所用，情为民所系，利为民所谋。坚持“两个务必”，继承和发扬艰苦奋斗优良传统。树立过紧日子思想，优化财政支出结构，大力压缩会议费、招待费、培训费、电话费、车船费、出国费等各项支出，确保公教人员工资、社会保障、农科教等重点支出需要。加强公务员队伍建设，落实廉政建设责任制，认真纠正部门和行业不正之风，从源头上治理和预防腐败。强化领导干部廉洁自律意识，坚持领导干部述职述廉制度，加大对领导干部的行政监察力度，严肃查处各类违纪违法案件。切实关心群众生活，加强信访督办和市民投诉受理工作，妥善处理突发性事件，确保社会稳定。

各位代表，振兴鞍山是全市人民多年的企盼，重铸辉煌是我们孜孜以求的目标。新的形势孕育新的希望，新的事业开启新的征程，让我们高举邓小平理论伟大旗帜，认真实践“三个代表”重要思想，万众一心，奋勇争先，与时俱进，开拓创新，为全面振兴鞍山老工业基地而努力奋斗！
